# The Association of Antipsychotic Postponement With 5-Year Outcomes of Adolescent First-Episode Psychosis

**DOI:** 10.1093/schizbullopen/sgad032

**Published:** 2023-11-14

**Authors:** Tomi Bergström, Tapio Gauffin

**Affiliations:** Department of Psychiatry, The Wellbeing Services County of Lapland, Kemi, Finland; Department of Psychology, University of Jyväskylä, Jyväskylä, Finland; Department of Strategic Services, The Wellbeing Services County of Lapland, Rovaniemi, Finland

**Keywords:** effectiveness, neuroleptics, psychotropics, pediatrics, schizophrenia

## Abstract

**Background and Hypothesis:**

Based on the need-adapted approach, delaying antipsychotics could help identify first-episode psychosis (FEP) adolescents who might not require them. However, some individuals might need antipsychotics, and postponing could harm their prognosis. This nationwide register-based follow-up aimed to test these two hypotheses.

**Study Design:**

All adolescents aged 13–20 with a psychotic disorder (ICD-10 codes: F20–F29) in Finland between 2003 and 2013 were identified (*n* = 6354) from national registers. For each case, a fixed 1825-day follow-up period was established from the onset of psychosis or until death. The outcome was considered “good” if adolescents did not die and had not received psychiatric treatment and/or disability allowances during the final year of follow-up. Testing the first hypothesis involved all antipsychotic treatment-naïve adolescents with FEP (*n* = 3714). The second hypothesis was tested with a sub-sample of only those who had received antipsychotics during follow-up (*n* = 3258). To account for baseline confounders, hypotheses were tested via a stabilized inverse probability of treatment weighted generalized linear models with logit link function.

**Study Results:**

Immediate antipsychotic treatment after the onset of psychosis was associated with poor 5-year outcome (adjusted odds ratio [aOR]: 1.8, 95% CI: 1.6–2.1). There was no statistically significant association between antipsychotic postponement and treatment outcome in those who eventually received antipsychotic treatment (aOR: 1.02, 95% CI: 0.7–1.2, *P*: .8), thus not providing support for second hypothesis.

**Conclusions:**

There is a significant subgroup of adolescent with psychosis who do not require immediate antipsychotic treatment. A more robust design is needed to evaluate the causality of the observed association.

## Introduction

Antipsychotic medications play a central role in both the acute and long-term management of psychotic disorders.^1,2^ While antipsychotic medications often reduce the intensity of psychotic symptoms, the long-term risk-benefit ratio of antipsychotic treatment remains unclear.^3,4^ In several registry studies, non-medication periods have been associated with an increased risk of relapse and mortality among patients diagnosed with schizophrenia.^5–7^ However, the design of these studies has faced criticism^8–10^ because real-world settings involve multiple factors that may simultaneously affect medication discontinuation and outcomes. Moreover, in many longitudinal studies higher cumulative exposure to antipsychotics have been associated with poorer outcome in the treatment of schizophrenia and other non-affective psychoses.^11–17^ Despite the attempts to control for confounding effects, it’s likely that some of these results are due to the confounding by indication, as those with more severe symptoms are more likely receiving antipsychotics.^11^

Despite limited research evidence, early initiation of antipsychotic medication has been considered important for improving long-term outcomes in so called early intervention approaches.^18^ This is based on observations that longer durations of untreated psychosis are associated with poorer outcomes^19,20^ and the hypothesis that antipsychotics are triggering a remedial process of neuroprogression.^18^ However, there is no support for hypothesis that active psychosis itself is a neurotoxic stage,^21,22^ automatically leading to a deteriorated course that requires antipsychotics.

More recent research has not supported idea on the preventive usage of antipsychotics as a first-line treatment with young people in ultra-high risk for psychosis.^23^ There is also recent controlled evidence suggesting that in the treatment of first-episode psychosis (FEP), antipsychotics may not be necessary if immediate psychosocial support is provided.^24,25^ An earlier example of this kind of a practice is the need-adapted approach developed in Finnish mental health services,^26^ where antipsychotic medication was only used if the situation prolonged or escalated during the psychotherapeutically-oriented treatment process. Quasi-experimental research^27^ suggested that need-adapted treatment response together with 1-month postponement of antipsychotic medication helped to identify FEP patients who do not require antipsychotics, and was generally associated with more favorable long-term outcomes compared to standard antipsychotic treatment for FEP.

Overall, the mixed outcomes of antipsychotic treatment in patients diagnosed with psychosis can be partly attributed to validity issues within the current diagnostic system. This is because individuals classified under the psychotic disorders are highly heterogeneous on symptoms and causes and may therefore respond differently to antipsychotic treatment.^28^ Additionally, the timing of treatment in relation to the first episode of psychosis may also impact the outcomes of antipsychotic treatment. Given that psychosis typically emerges in adolescence or early adulthood, there is growing interest in early intervention strategies aimed for young people with psychotic symptoms. It is important to gain more insight into the course and role of antipsychotic treatment strategies in this population, as adolescents may be especially vulnerable to developing adverse medication effects.^23,29^

Based on research on the need-adapted approach,^16,27,30,31^ it is plausible that 1-month delaying the administration of antipsychotic medication can help identify first-episode patients who may not require antipsychotic treatment. Since this reduce the risk of iatrogenic physiological and psychological medication effects, our first hypothesis was that a 1-month postponement of antipsychotics after adolescent FEP would be associated with improved long-term outcomes. However, it is also plausible that a subgroup of patients may require antipsychotic medication, and delaying its administration may negatively affect their long-term outcomes. Therefore, our second hypothesis posited that delaying medication would be associated to poorer long-term outcomes in adolescents who eventually required antipsychotics. To further investigate these two hypotheses, this study utilized a nationwide longitudinal register-based cohort design involving a sample of adolescents with FEP.

## Methods

### Research Cohorts

**Fig. 1.**
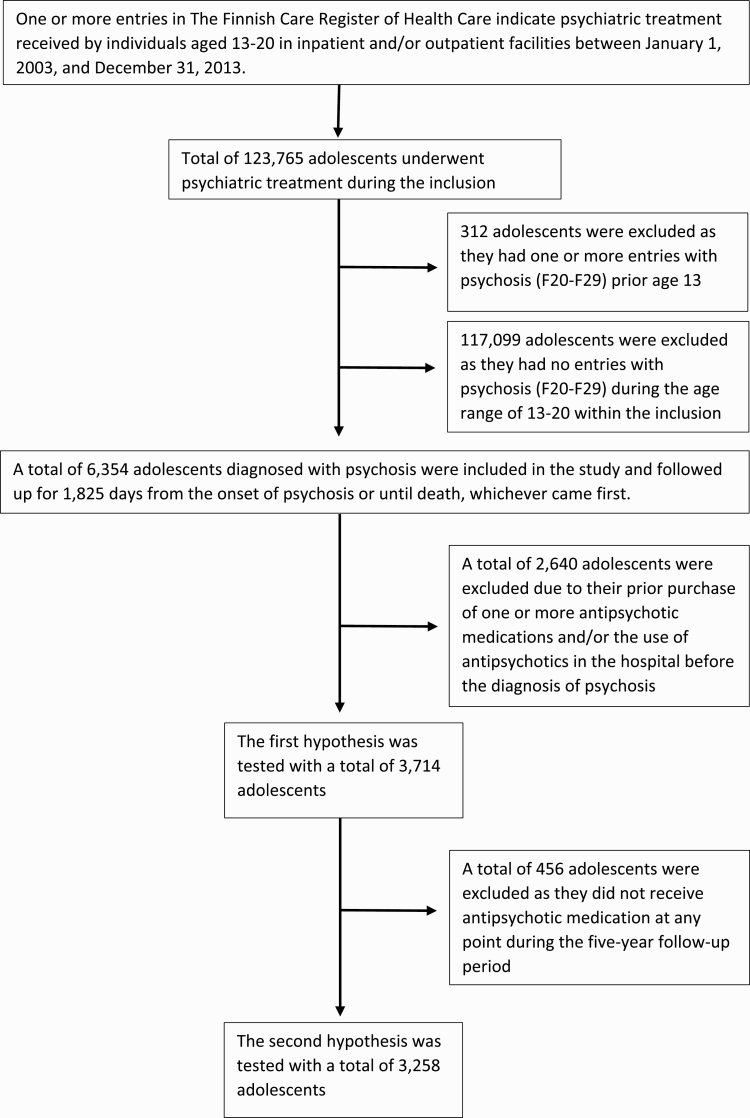
The flow of cases from the total cohort into the study and the two hypothesis testing sub-samples.

The research cohort was formed from a larger cohort^32,33^ of all adolescents aged 13–20 who received psychiatric treatment in Finland in 1.1.2003–31.12.2013 (*n* = 123 765). Data was collected until the end of 2018, enabling a continuous 5-year register-based follow-up for all cases in the cohort. From this cohort, adolescents with one or more registered entry with a diagnosis of psychotic disorder (ICD-10-codes: F20–F29) were identified (*n* = 6354) (figure 1). The onset of psychosis was defined as the date of the first registered entry with a psychosis. For each cases, the fixed follow-up period was set at 1825 days (5 years) from the onset of psychosis or until the date of death, whichever occurred first.

A sub-sample of antipsychotic treatment-naïve adolescents was formed to test the first hypothesis, which aimed to determine whether a 1-month postponement of antipsychotic medication after the first episode of psychosis would associate to improved long-term outcomes.

To test the second hypothesis, another sub-sample was formed consisting of adolescents who had received antipsychotics during the follow-up. This sub-sample aimed to examine whether postponing medication would be associated with poorer long-term outcomes in those adolescents who eventually required antipsychotics.

### Measurements

Baseline-, treatment-, and outcome-variables were created by combining information from national social and healthcare registers. Baseline variables that were included as covariates in the statistical analyses were sex, age at the onset of psychosis, prior mental health treatment, prior psychiatric hospitalizations, prior anxiolytic treatment, prior antidepressant treatment, prior antipsychotic treatment, time to psychosis from the onset of adolescent mental health treatment, child protective services involvement prior to the onset of psychosis, hospitalization at the onset of psychosis, and F-diagnoses prior to psychosis. Since in Finland, the eastern and northern parts of the country had higher prevalence and incidence rates of psychotic disorders, possibly due to sociodemographic and genetic factors,^34^ this categorization was also included as one of the baseline variables. Given the cautious approach towards diagnosing schizophrenia in adolescents, for the purpose of evaluating the severity and chronicity level of FEP, adolescents were considered to have schizophrenia if there was at least one entry with the F20-code within the first year of follow-up. This approach takes into account the possibility of delayed or evolving schizophrenia diagnoses.

The independent exposure variable was the postponement of antipsychotic medication after the onset of psychosis. Antipsychotics were defined as postponed if there were no purchases of antipsychotics (ACT-code: N05A excluding lithium) and/or antipsychotics were not used in the hospital within the first month from the first diagnosis of psychosis.

The outcome was considered “good” if adolescents did not die by the end of the 5-year follow-up and had not received any form of psychiatric treatment, supportive housing, or disability allowances during the last year of follow-up. This criterion can be seen as analogous to symptomatic recovery because in Finland, services and disability allowances are provided to the entire population based on national social insurance. Therefore, it is highly unlikely that an individual would have no registered entries at all over a long period of time if their psychiatric symptoms remained disabling.

### Statistical Analysis

Differences in baseline binominal characteristics between the outcome and intervention groups were compared using the chi-square test. The normality of the continuous baseline variables (age, time to psychosis, and time to death) was assessed using the Kolmogorov–Smirnov test. As the assumption of normality was found to be violated, the differences in the continuous variables were compared via a non-parametric Mann–Whitney *U*-test. To detect confounding effects to good and poor outcomes, association of baseline characteristics with the outcome of interest was analyzed using a multivariable logistic regression analysis including all baseline variables.

Prior to hypothesis testing, observable differences in baseline characteristics between the immediate antipsychotic treatment group and the antipsychotic postponement group were adjusted using stabilized inverse probability of treatment weighting (SIPTW)^35^ to control for confounding by indication. Propensity scores were calculated using a multivariable logistic regression, with the treatment group variable as the dependent variable and all baseline variables as independent variables. Propensity scores were then used to calculate stabilized inverse probability of treatment weights for each case.

Hypotheses on antipsychotic treatment postponements associations on long-term outcomes were tested using weighted generalized linear models with a binomial probability distribution and a logit link function. Adjusted odds ratios (aOR) were used to assess the direction and effect size of antipsychotic postponement on a good outcome within two sub-samples. *P* values below .05 were considered statistically significant. Sensitivity analyses with the *E*-value^36^ were conducted to examine the extent to which unmeasured confounders would need to exist to render a significant ratio above 1 as non-significant.

All analyses were conducted using SPSS 28 for Windows.

## Results

### Group Characteristics

The average annual incidence of FEP during the inclusion years was 99 per 100 000 (95% CI: 92–106) adolescents of similar age. The average age of onset of adolescent FEP was 17.4 years (SD: 2), with 51% being female and 73% having prior psychiatric treatment. The average time from onset of adolescent psychiatric treatment to psychosis was 524 days (standard deviation [SD]: 679 days).

A total of 1475 (23%) adolescents were defined as demonstrating a good outcome since they survived until the end of the 5-year follow-up period and no longer received any treatment or disability allowances for mental health problems (table 1). In the multivariable regression model, statistically significant baseline predictors (*P* < .05) for a poor outcome were a diagnosis of schizophrenia (OR: 3.6, 95% CI: 2.8–4.6), prior antipsychotic treatment (OR: 1.6, 95% CI: 1.3–1.8), hospital admission at onset of psychosis (OR: 1.5, 95% CI: 1.3–1.9), a prior diagnosis of anxiety disorder (F4) (OR: 1.4, 95% CI: 1.2–1.7), and prior antidepressant treatment (OR: 1.3, 95% CI: 1.1–1.5).

**Table 1.** Baseline Demographic and Clinical Characteristics of Good and Poor Long-Term Outcome of Adolescent Patients With First-Episode Psychosis

	Good Outcome *n* = 1475	Poor Outcome *n* = 4879	*P*
Sex, women	790 (54%)	2452 (50%)	.026
Age (mean [sd])	17.3 (2)	17.5 (2)	<.001
North/East Finland	371 (25%)	1273 (26%)	.471
Prior child protective services	267 (18%)	938 (19%)	.335
Prior psychiatric treatment	995 (68%)	3644 (75%)	<.001
Time from treatment to FEP in days (mean [sd])	404 (606)	560 (696)	<.001
Prior diagnoses			
No F-diagnosis	679 (46%)	1776 (36%)	<.001
F1	70 (5%)	273 (6%)	.21
F3	423 (29%)	1780 (36%)	<.001
F4	273 (19%)	1319 (27%)	<.001
F5	50 (3%)	222 (5%)	.054
F6	27 (2%)	201 (4%)	<.001
F7	0	49 (1%)	<.001
F8	57 (4%)	286 (6%)	.003
F9	285 (19%)	1105 (23%)	.007
Schizophrenia-diagnosis during the first year after FEP	73 (5%)	840 (17%)	<.001
Antipsychotics prior FEP	454 (31%)	2186 (45%)	<.001
Hospital treatment prior FEP	951 (65%)	3622 (74%)	<.001
Hospital treatment at onset of FEP	531 (36%)	2293 (47%)	<.001

*Note*: FEP, first episode psychosis; SD, standard deviation.

A total of 3714 (58%) adolescents were antipsychotic treatment-naïve at the onset of psychosis and were thus included in the primary analyses. Among them, 1549 (42%) did not receive antipsychotics within the first month from the onset of psychosis. Most significant predictor for immediate antipsychotic treatment was hospitalization at the onset of psychosis (table 2). The group whose antipsychotic treatment was postponed was also younger and less likely to be diagnosed with schizophrenia or to have received prior hospital treatment. However, the proportion of adolescents with previous treatment contact and a longer time from the onset of psychosis, as well as a higher proportion of prior mood, psychological development, and behavioral disorders, was higher in those whose antipsychotics were postponed after the onset of psychosis. This indicates that they were more likely to already have mental health treatment contact due to other types of problems.

**Table 2.** Group Characteristics of Antipsychotic Postponement vs Immediate Antipsychotic Treatment Group Prior and After the Weighting (Antipsychotic Treatment-Naive Cases)

	Non-weighted Sample			Inverse Probability of Treatment Weighted Sample		
	Antipsychotic Postponement (*n* = 1549)	No Postponement (*n* = 2165)	*P*	Antipsychotic Postponement (*n* = 1490)	No Postponement (*n* = 2195)	*P*
Sex, women	824 (54%)	1178 (54%)	.464	804 (54%)	1196 (55%)	.752
Age (mean [sd])	17.2 (2)	17.4 (2)	.012	17.1 (2)	17.2 (2)	.389
North/East Finland	418 (27%)	571 (26%)	.678	371 (25%)	581 (27%)	.280
Child protective services	231 (15%)	288 (13%)	.163	196 (13%)	296 (14%)	.721
Prior psychiatric treatment	980 (63%)	1249 (58%)	<.001	877 (59%)	1295 (59%)	.993
Hospital admission at onset	135 (9%)	1752 (81%)	<.001	727 (49%)	1099 (50%)	.447
Time in days from treatment to FEP (mean [sd])	392 (620)	319 (564)	<.001	337 (559)	338 (578)	.231
Prior diagnoses						
No diagnosis	775 (50%)	1229 (57%)	.001	878 (53%)	1203 (54%)	.235
F1	51 (3%)	76 (4%)	.719	48 (3%)	74 (3%)	.800
F3	385 (25%)	463 (21%)	.013	317 (21%)	479 (22%)	.692
F4	286 (19%)	348 (16%)	.056	255 (17%)	372 (17%)	.895
F5	47 (3%)	57 (5%)	.465	37 (3%)	54 (3%)	.967
F6	32 (2%)	28 (1%)	.066	17 (1%)	33 (2%)	.351
F7	8 (0.5%)	11 (0.5%)	.972	9 (1%)	12 (1%)	.836
F8	70 (5%)	53 (2%)	.001	56 (4%)	84 (4%)	.912
F9	282 (18%)	293 (14%)	.001	274 (18%)	351 (16%)	.121
Schizophrenia-diagnosis during the first year after FEP	73 (5%)	840 (17%)	.001	193 (13%)	288 (13%)	.876
Hospital treatment prior FEP	800 (52%)	1920 (89%)	.001	1082 (73%)	1551 (71%)	.154
Anxiolytics prior FEP	137 (9%)	216 (10%)	.246	130 (9%)	203 (9%)	.586
Antidepressants prior FEP	282 (25%)	382 (27%)	.106	366 (25%)	550 (25%)	.725
Medication after FEP						
Anxiolytics	447 (29%)	793 (37%)	.001	445 (30%)	726 (33%)	.039
Antidepressants	849 (55%)	1228 (57%)	.247	738 (49%)	1269 (58%)	<.001
Antipsychotics (after first follow-up year)	863 (56%)	1703 (79%)	.001	851 (57%)	1687 (77%)	<.001
Five-year outcome						
Treatment contact	786 (51%)	1310 (61%)	<.001	760 (51%)	1295 (59%)	<.001
Psychotropics	779 (50%)	1448 (67%)	<.001	732 (49%)	1403 (64%)	<.001
Disability allowances	473 (31%)	946 (44%)	<.001	396 (27%)	886 (40%)	<.001
Death	29 (2%)	45 (2%)	.657	16 (1%)	41 (2%)	.055
Days to death from FEP (mean [sd])	919 (545)	704 (502)	.043	909 (557)	823 (540)	.374
Good outcome	545 (35%)	476 (22%)	<.001	544 (37%)	528 (24%)	<.001

*Note*: FEP, first episode psychosis; SD, standard deviation.

In the antipsychotic postponement group, more adolescents demonstrated a good outcome. There were no significant differences in mortality. After applying SIPT-weighting, there were no longer differences in baseline characteristics, and the outcomes remained the same (table 2).

Out of the 1549 adolescents whose antipsychotics were postponed, 456 (29%) did not receive antipsychotics at any point during the 5-year follow-up and were excluded from testing the hypothesis regarding whether antipsychotic postponement is associated with a declined outcome in those who eventually require antipsychotics. The baseline differences remained similar as described above (table 3). There were no significant differences in the proportion of good outcomes or the total mortality rate between the exposure groups of those who eventually required antipsychotics. However, the probability of disability allowances at the end of the follow-up was significantly higher and time to death shorter for those with immediate antipsychotic medication as compared to those whose antipsychotic medication was postponed. The results remained the same after applying SIPT-weighting (table 3).

**Table 3.** Group Characteristics of Antipsychotic Postponement vs Immediate Antipsychotic Treatment Group Prior and After Weighting (Only Cases With Antipsychotic Usage After Onset of Psychosis)

	Non-weighted Sample			Inverse Probability of Treatment Weighted Sample		
	Antipsychotic Postponement (*n* = 1093)	No Postponement (*n* = 2165)	*P*	Antipsychotic Postponement (*n* = 1051)	No Postponement (*n* = 2178)	*P*
Sex, women	574 (53%)	1178 (54%)	.306	568 (54%)	1175 (54%)	.938
Age (mean [sd])	17.3 (2)	17.4 (2)	.229	17.1 (2)	17.3 (2)	.146
North/East Finland	289 (26%)	571 (26%)	.967	265 (25%)	581 (27%)	.372
Child protective services	158 (15%)	288 (13%)	.366	291 (13%)	291 (13%)	.527
Prior psychiatric treatment	983(63%)	1249 (58%)	.008	630 (60%)	1306 (60%)	.928
Hospital admission at onset	98 (9%)	1752 (81%)	<.001	577 (55%)	1229 (57%)	.421
Time from treatment to FEP (mean [sd])	395 (623)	319 (564)	<.001	332 (547)	341 (575)	.334
Prior diagnoses						
No diagnosis	547 (50%)	1229 (57%)	<.001	573 (54%)	1178 (54%)	.827
F1	39 (4%)	76 (4%)	.933	34 (3%)	75 (3%)	.757
F3	463 (25%)	463 (21%)	.018	214 (20%)	480 (22%)	.274
F4	207 (19%)	348 (16%)	.040	184 (17%)	376 (17%)	.855
F5	33 (3%)	57 (5%)	.525	24 (2%)	56 (3%)	.625
F6	19 (2%)	28 (1%)	.314	10 (1%)	31 (1%)	.261
F7	8 (1%)	11 (0.5%)	.428	7 (1%)	14 (1%)	.938
F8	48 (4%)	53 (2%)	.003	45 (4%)	76 (3%)	.268
F9	202 (19%)	293 (14%)	<.001	184 (17%)	335 (15%)	.122
Schizophrenia-diagnosis during the first year after FEP	130 (12%)	840 (17%)	<.001	150 (14%)	328 (15%)	.551
Hospital treatment prior FEP	608 (56%)	1920 (89%)	<.001	805 (77%)	1689 (77%)	.576
Anxiolytics prior FEP	108 (10%)	216 (10%)	.931	88 (8%)	214 (10%)	.183
Antidepressants prior FEP	293 (27%)	382 (27%)	.897	244 (23%)	571 (26%)	.065
Medication after FEP						
Anxiolytics	406 (37%)	793 (37%)	.873	379 (36%)	741 (34%)	.246
Antidepressants	699 (64%)	1228 (57%)	<.001	610 (58%)	1255 (58%)	.799
Antipsychotics (after first follow-up year)	863 (56%)	1703 (79%)	<.001	811 (77%)	1680 (77%)	.966
Five-year outcome						
Treatment contact	1310 (63%)	1310 (61%)	.177	649 (62%)	1287 (60%)	.153
Psychotropics	1448 (65%)	1448 (67%)	.347	657 (63%)	1409 (65%)	.233
Disability allowance	439 (40%)	946 (44%)	.054	352 (33%)	895 (41%)	<.001
Death	22 (2%)	29 (2%)	.901	11 (1%)	40 (2%)	.091
Days to death from FEP (mean [sd])	1028 (544)	704 (502)	.019	1028 (544)	704 (502)	.019
Good outcome	229 (21%)	476 (22%)	.498	252 (24%)	513 (24%)	.796

*Note*: FEP, first episode psychosis; SD, standard deviation.

### Outcome

In antipsychotic treatment-naïve adolescent FEP patients, immediate antipsychotic treatment after the onset of psychosis was associated with at statistically significant level on poor 5-year outcome (adjusted odds ratio [aOR]: 1.8, 95% CI: 1.6–2.1, *P* < .001). *E* values indicated that it would require moderate-to-substantial residual confounding to render the findings non-significant (for point estimate: 2.02 and for CI: 1.84). There was no statistically significant association between antipsychotic postponement and treatment outcome in those who eventually received antipsychotic treatment (aOR: 1.02, 95% CI: 0.7–1.2, *P*: .807), thus not providing support for second hypothesis.

## Discussion

In this nationwide register-based 5-year follow-up study, which included all Finnish adolescents with FEP between 2003 and 2013, it was found that immediate antipsychotic medication was associated with a poorer 5-year outcome compared to a 1-month antipsychotic postponement, after controlling for observable confounders. These findings align with previous research on need-adapted approaches^16,26,27,31^ and recent controlled trials,^24,25^ suggesting that there is a significant subgroup of patients with acute psychosis who do not require immediate antipsychotic treatment, and postponing medication, particularly in first-episode cases, may help identify those patients.

Since there may still be a subgroup of psychosis patients who require immediate or preventive antipsychotic treatment to prevent a deteriorated course of FEP, it was hypothesized that postponement of antipsychotics for those who eventually used antipsychotics would be associated with poor outcomes. However, findings did not support this hypothesis. There was no indication that antipsychotics initiated prior to the formal diagnosis of psychosis or immediate antipsychotic treatment for those who eventually required antipsychotics would improve treatment outcomes. On the contrary, antipsychotic treatment prior to psychosis was associated with a poorer long-term outcome, but this may be partially due to confounding by indication, where individuals with more severe symptoms receive antipsychotics even without a formal diagnosis. Nevertheless, after adjusting for evident confounders such as prior medication, hospitalizations, and schizophrenia diagnosis, antipsychotic postponement was not significantly associated with a poorer long-term outcome and increased mortality. In fact, contrary to our hypothesis, the time to death after FEP was found to be significantly longer for individuals whose antipsychotic medication was postponed. Furthermore, after controlling for confounding factors in both sub-samples, immediate antipsychotic medication was associated with a notably higher disability ratio at the end of the 5-year follow-up period. These findings suggest that the assumption of diagnosable non-affective psychotic disorder being a neurodegenerative disease,^37,38^ which necessitates immediate antipsychotic treatment for improved functional outcomes, may not hold true.

It should be noted that above findings may not be generalized outside of adolescent first-episode patients and due to the observational nature of study, no causal relationships can be drawn. There are nevertheless some potential explanations for these observations that would merit further attention in future studies. First, the term psychosis refers to a wide variety of different mental states, when the general assumption that diagnosis can be reduced to certain common neurobiological causes that antipsychotic treatment corrects may be invalid in the first-place.^39^ It has been proposed that the symptom-reducing effect of psychotropic can be the direct consequence of a more non-specific factors of medication treatment, such as sedation, apathy, placebo, and reduction of difficult emotions.^40^ While this kind of a symptom reduction may reduce harmful social and other risks associated with acute psychosis, prolonged antipsychotic treatment may simultaneously increase the risk of previously documented iatrogenic effects, such as brain volume changes,^41^ anticholinergic,^42^ and other physiological^43^ and psychological side-effects,^43,44^ which may eventually outweigh the benefits. Moreover, prolonged antipsychotic treatment may also increase the risk of medication withdrawal symptoms, which could contribute to self-fulfilling prophecies.^45^ These factors would explain the findings of this study as well as the previous mixed results from observational studies, but they still merit’s further research with more robust design.

### Strength and limitations

Finnish registers are recognized as reliable sources of information,^46^ enabling the non-selective inclusion of all adolescents who received treatment for FEP within the specified years. The annual incidence of diagnosable psychotic disorders was aligned with recent data on incidence of psychosis among adolescents and young adults,^47^ indicating valid recognition of psychosis at this age group. These factors, including also the ability to facilitate continuous follow-up without any loss of cases, collectively contribute to minimizing sampling-related ascertainment bias.

However, it is important to exercise caution when interpreting the main findings due to a certain ascertainment bias relating to the lack of randomization. The most significant limitation of our real-world study design lies in our inability to gather comprehensive information on the reasons behind the delay in administering antipsychotic treatment. One observable confounding factor in our analysis was the initial severity of the condition, as individuals with more severe disorders were more inclined to receive immediate antipsychotic treatment, thus introducing a confounding by indication-bias. However, it is worth noting that we successfully adjusted our primary analysis for obvious severity factors such as hospital treatment, prior diagnostic distributions and a diagnosis of schizophrenia without encountering significant instability in the weighting, suggesting that other factors also contribute to the postponement of antipsychotic treatment. This reduces the potential for confounding by indication when utilizing weighted generalized linear models.

Besides symptom severity, potential reasons for postponement could be related to variations in treatment orientations and practices among healthcare services and singular professionals, including a general carefulness in starting long-term medication for adolescents. Moreover, it is possible that some patients in antipsychotic postponement group had antipsychotic prescription, but they never purchased their medication. Conversely, it is also possible that some adolescents purchased antipsychotic medication without actually using it. However, we believe it is unlikely that there is a systematic pattern of adolescents purchasing antipsychotics without any usage, and unfilled first-month prescription do not affect the main conclusion of this research, since it is clear indication that antipsychotics were not used.

## Conclusions

The longitudinal register study suggests that delaying antipsychotic medication for 1 month in Finnish adolescents with FEP associated to better long-term outcomes compared to immediate antipsychotic treatment. However, caution is needed when interpreting the findings due to residual confounding and the lack of information on reasons for medication postponement. Despite these limitations results showed that there is significant subgroup of adolescents who have fulfilled diagnostic criteria of psychotic disorder, and whose antipsychotic medication has been postponement for 1 month without significant increase of adverse outcomes as compared immediate antipsychotic usage. To ascertain causality for the observed association, there is need for a more robust research design that includes randomization.
